# Editorial: Is autism a biological entity?

**DOI:** 10.3389/fpsyt.2023.1180981

**Published:** 2023-05-02

**Authors:** Lynn Waterhouse, Laurent Mottron

**Affiliations:** ^1^Child Behavior Study, The College of New Jersey, Ewing Township, NJ, United States; ^2^Department of Psychiatry, Montreal University, Montreal, QC, Canada

**Keywords:** autism, diagnosis, endophenotypes, prototypes, ASD

There has been no single cause or pathophysiology found to be unique to all those with autism, but current diagnostic criteria are linked to nearly two hundred genetic and environmental reported causes. The current DSM-5 criteria for an autism *spectrum* diagnosis (ASD) allow hundreds of varied patterns of persistent deficits in social communication and social interaction, and myriad patterns of restricted and repetitive activities and interests. This wide phenotypical heterogeneity –which appears to have markedly increased in the two last decades–has led many researchers to question the validity of the ASD diagnosis.

The papers in this special section explore varied aspects of the relationship between the many biological causes of autism and the heterogeneity of diagnostic symptoms and comorbidities. None of the twelve papers support the DSM-5 ASD criteria as defining a unitary biological entity or natural kind. Instead, the twelve papers include proposals to disentangle autism from ASD by adopting a new autism diagnosis, or by reducing the heterogeneity of causes and symptoms linked to autism, or by establishing a new causal model of autism. Taken together, all these papers assert that the heterogeneity of symptoms and causes is a core problem for autism research, and each paper views the current ASD criteria as an impediment to the discovery of meaningful categories of neurodevelopmental disorders.

## Proposals for new diagnosis of autism

Crespi while underlining the issues associated with current study of autism, particularly over-inclusivity, proposed that a new autism diagnosis should be based on the recognition of a clinical pattern combining Mottron's (1) prototypical autism and Kanner's “hallmarks” of autism. Mottron outlined a new diagnosis of autism in which expert clinicians would agree on a more limited set of autism criteria in an autism sample homogeneous for comorbidity, language problems, intelligence, age, and sex. Kanner's hallmarks include extreme aloneness, severe language deficits, good cognition, intense focus on objects, repetitive behaviors, and insistence on sameness.

Like Crespi, Green argued that Mottron's model of prototypical autism should be the categorical baseline for understanding autism. However, Green proposed that ‘autism states' reflect both the emergence and subsidence of the autism phenotype. There would be three ways of studying autism states: through clinical descriptions or longitudinal observations of the emergence of phenotypes in early development; through clinical descriptions or longitudinal observations of the subsidence of phenotypes of autism later in development; and through the study the emergence of autism states by means of experimental interventions in autism development. Green outlined his research program of experimental interventions, and argued that intervention studies offer the most rigorous means to test the phenomenon of emergence because they provide a controlled test of developmental change.

Fernell and Gillberg noted that early diagnoses of neurodevelopmental disorders overlap and change with time. They advocated for an umbrella category located higher in a taxonomic hierarchy, ESSENCE, Early Symptomatic Syndromes Eliciting Neurodevelopmental Clinical Examinations. ESSENCE would regroup early manifestations of childhood disorders that include impairments in motor, cognitive, neurological, communicative and social development, as well as sleep, feeding and behavioral regulation. The ESSENCE group identifies the very high rate of “comorbidities” in childhood disorders, and assumes developmental deviations or delays in speech and language and motor development are unspecific.

Hens and Van Goidsenhaven suggested that developmental diversity should be the starting point for research, rather than a static categorical autism diagnosis. They argued that interaction with environment moves the categorical boundary. A developmental diversity approach could clarify comorbidities, and enrich genes-based research without starting from diagnostic categories. They have advocated a neurodiversity-sensitive /translational perspective, wherein autism research should include children and adults who may not receive a diagnosis but who may be diverse in symptoms and causes.

Lombardo and Mandelli reviewed the history of the autism diagnostic criteria, emphasizing how the role of language level and developmental history have been gradually lessened in successive DSM criteria for autism. They asserted that the current DSM-5 criteria are only optimized to be sensitive and specific for the differentiation of autism vs. non-autism. These criteria are not valid for explaining autism biology, outcomes, and treatment response (BOT). Researchers should develop a variety of new diagnostic definitions or models of autism to address BOT. Creating varied new diagnoses does not mean the current autism diagnosis has failed, because it is still valid for maximizing clinical consensus based on autism behavior.

Phillipe maintained her confidence in a categorical diagnosis of autism. However, she claimed that studying autism should primarily identify the features that are unique to the individual. Standardized autism diagnoses should only be conducted after individual variation is identified. She asserted that syndromic autism–where a specific genetic or other specific cause is known– approaches the definition of a natural kind by means of the detection of unique sets of clinical features.

## Proposals for resolving heterogeneity in autism

Eigsti and Fein argued that the heterogeneous causes can best be resolved by creating smaller homogeneous groups formed by clinical DSM 5 Specifiers: IQ, language, and outcome status. Compared to autistic and non-autistic groups, Children who had lost their autism diagnosis (LAD) have a pattern of language-related brain activations similar to that found in the autistic individuals, but also had many brain activations that were unique to LAD. Their findings demonstrate how biomarkers can be orthogonal to longitudinal trajectories.

Levy noted that neurobiological research does not support a categorical definition of ASD, and argued that a reconceptualization of ASD is needed but could only occur when there is profound dissatisfaction with the diagnosis among clinical and research communities as well as stakeholders.

Loth stated that efforts to divide autism into subgroups by biomarkers such as brain structures have not yet identified any clearly delineated diagnostic subgroups. Loth recommended that future research address the problem of the additive and interactive effects between biological and social mechanisms, while focusing on finding transdiagnostic groups of individuals across neurodiverse populations.

Waterhouse underlined the failure of iterative DSM attempts to reduce autism heterogeneity. She underlined the current inability to map biological causes to distinctly categorized phenotypes. From this, and from the variability in symptom presentation and development, she questioned the unity of autism as a *biological* entity. She argued that autism heterogeneity may be addressed by the discovery of transdiagnostic neurodevelopmental groups, grounded on endophenotypes.

## Proposals for a new causal model of autism

Chawner and Owen proposed that autism is the result of two biological dimensions that combine to yield individual variation: a population-wide continuum of social and adaptive functioning resulting from multiple alleles of small effect, and a continuum of childhood-onset disorders such as intellectual disability (ID) and attention deficit/hyperactivity disorder (ADHD), and adult-onset schizophrenia and bipolar disorder linked to *de novo* genetic mutations. Commenting their proposition, Sarovic argued that varied types of disorders stem from the magnitude of rare genetic risk. He rather proposes a three-factor model of autism: natural variation in non-pathological traits, a range of neurodevelopmental risks, and adaptive behaviors that moderate the links between the first two factors.

A consensus seems to arise from these empirical and theoretical positions. The current ASD criteria are ineffective, and the use of these criteria has not yet led to convincing discoveries. Nonetheless, whether the ASD criteria should still be used as a basis for research remains an open question. Consequently, research independent of DSM-5 ASD criteria that adopts a new autism diagnosis such as prototypes, or explores a new causal model of autism, or develops transdiagnostic endophenotypes, must be encouraged.

## Author contributions

Both authors listed have made a substantial, direct, and intellectual contribution to the work and approved it for publication.

## References

[1] Mottron L. A radical change in our autism research strategy is needed: back to prototypes. Autism Res. (2021) 14:2213–20. 10.1002/aur.2494 34077611

